# The natural place to begin: The ethnoprimatology of the Waorani

**DOI:** 10.1002/ajp.22173

**Published:** 2013-07-01

**Authors:** Sarah Papworth, EJ Milner-Gulland, Katie Slocombe

**Affiliations:** 1 Division of Ecology and Evolution, Imperial CollegeAscot, United Kingdom; 2 Centre for Environmental Policy, Imperial CollegeLondon, United Kingdom; 3 Department of Psychology, University of YorkYork, United Kingdom

**Keywords:** ethnobiology, Amazon, Yasuní National Park, ecological knowledge, *Lagothrix*

## Abstract

Ethnoprimatology is an important and growing discipline, studying the diverse relationships between humans and primates. However there is a danger that too great a focus on primates as important to humans may obscure the importance of other animal groups to local people. The Waorani of Amazonian Ecuador were described by Sponsel [Sponsel (1997) New World Primates: Ecology, evolution and behavior. New York: Aldine de Gruyter. p 143–165] as the “natural place” for ethnoprimatology, because of their close relationship to primates, including primates forming a substantial part of their diet. Therefore they are an ideal group in which to examine contemporary perceptions of primates in comparison to other types of animal. We examine how Waorani living in Yasuní National Park name and categorize primates and other common mammals. Although there is some evidence that the Waorani consider primates a unique group, the non-primate kinkajou and olingo are also included as part of the group “monkeys,” and no evidence was found that primates were more important than other mammals to Waorani culture. Instead, a small number of key species, in particular the woolly monkey (*Lagothrix poeppigii*) and white-lipped peccary (*Tayassu pecari*), were found to be both important in the diet and highly culturally salient. These results have implications for both ethnoprimatologists and those working with local communities towards broader conservation goals. Firstly, researchers should ensure that they and local communities are referring to the same animals when they use broad terms such as “monkey,” and secondly the results caution ethnoprimatologists against imposing western taxonomic groups on indigenous peoples, rather than allowing them to define themselves which species are important. Am. J. Primatol. 75:1117–1128, 2013. © 2013 The Authors. *American Journal of Primatology* Published by Wiley Periodicals, Inc.

## INTRODUCTION

Ethnoprimatology is a subiscipline of primatology which aims to understand the interconnections between humans and other primates, often with the ultimate aim of informing conservation [Fuentes & Hockings, 2010]. Ethnoprimatological studies contrast with traditional research on wild primates, in that humans are viewed as an integral part of the primate ecosystem, rather than a source of disturbance or “unnatural” behavior [Fuentes, [Bibr b17],[Bibr b18]; Riley, 2006]. Although historically the term ethnoprimatology has been used to describe this sub-discipline [originally suggested by Sponsel [1997], p. 145 “for lack of a better designation”], the name is confusing within the broader nomenclature in biology/social sciences. Ethnobiology is the study of human biological knowledge (encompassing the subdisciplines of ethnobotany and ethnozoology which study plants and animals respectively), including culture, linguistics, use and management, and even aspects of archeobiology and archeobotany [Anderson, 2011], thus including only some aspects of ethnoprimatology. In contrast, anthrozoology is the study of human relationships with non-human animals and includes both the subject area and methodologies of ethnoprimatology [Fuentes, 2012]. Although Fuentes [2012] argues that the “ethno” in ethnoprimatology has a different meaning in other ethno-disciplines such as ethnobiology, ethnoprimatology [*sensu* Fuentes, 2012] may be better considered anthroprimatology, with the term ethnoprimatology specifically reserved for the study of human primatological knowledge. In order to distinguish these two areas of study and maintain consistency with existing literature, this paper will use “ethnoprimatology *sensu stricto*” to refer to the study of human primatological knowledge, and “ethnoprimatology” to refer to the sub-discipline as defined by Fuentes [2012].

Interest in the relationships between humans and other primates is growing, particularly in the context of conservation [Estrada, 2004; Lee, 2010; Parathian & Maldonado, 2010], though researchers who conduct this type of research may be unaware of the sub-discipline of ethnoprimatology. When ethnoprimatology was first proposed by Sponsel [1997] as an important area for investigation, he suggested that

The natural place to begin is with those indigenous societies, such as the Waorani, for which monkeys are an important species in the diet [Sponsel, 1997, p. 159].

Although the Waorani of the Ecuadorian Amazon have been well studied and have a high profile internationally [Finer et al., 2009], no detailed research has been conducted on contemporary Waorani perceptions of primates and other animals. Some ethnographic accounts do document some aspects of Waorani culture which relate to primates. For example, the Waorani are reported to specialize in hunting monkeys and birds [Rival, 1996], Waorani women have been observed to breast-feed infant monkeys [Rival, 2007], and “monkey houses” were traditionally constructed close to the longhouse [Mondragon & Smith, 1997]. Yet these are anecdotal reports of single incidences, or reports of traditional Waorani culture. Although reports of the historic importance of primates to Waorani culture are important, contemporary relationships, perceptions, and interactions also are of interest for ethnoprimatology, and have greater relevance to conservation and development practice.

**Fig. 1:**
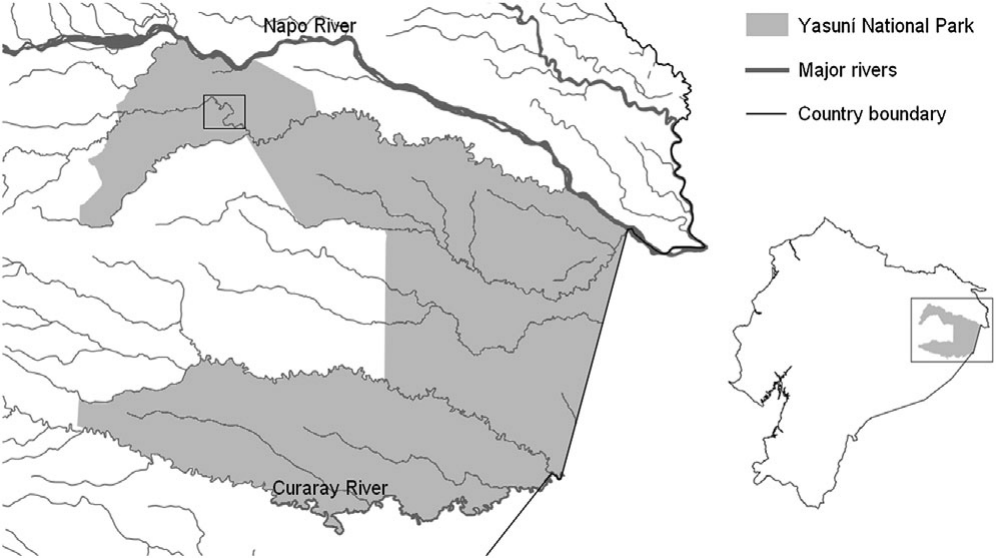
Location of study site in Ecuador and Yasuní National Park, indicated by the squares on each map.

The homeland of the Waorani is in Amazonian Ecuador, bounded to the north by the Napo River, and by the Curaray and Vilano Rivers to the south [Fig. 1, Cabodevilla, 1994]. This area is now part of Yasuní National Park and Waorani Territory, and the Waorani people have collection rights for all above ground resources [Finer et al., 2009], though it is illegal for these resources to be transported and sold outside the park. Traditionally, the Waorani were hunter-gatherer-farmers, growing a small number of cultivars in cleared forest, collecting wild plants, and hunting mostly large monkeys and peccaries [Rival, 2002]. Since first western contact in 1950, some Waorani have moved to permanent settlements, often centered on a school. Currently, there are around 2000 Waorani living in approximately 38 small scattered villages, and small groups scattered throughout the forest, pursuing more traditional ways of life [Beckerman et al., 2009; Finer et al., 2009; Lu, 2001]. Some communities living within Yasuní National Park and related to the Waorani (the Tagaeri and Taromenane) have refused all western contact. These communities pursue entirely traditional lifestyles [Finer et al., 2009], excepting limited integration of some western material goods, such as using plastic tape (found in abandoned oil facilities) on traditional spears [Proaño García & Colleoni, 2008].

Waorani hunting is still predominately for subsistence; Franzen [2006] estimated that only around 4% of all wildmeat extracted by three communities in the north of Yasuní National Park is sold at a local market outside the park by the Napo River. Since first western contact, however, many Waorani have changed their hunting methods from traditional spears and blowpipes to guns and dogs [Franzen, 2006; Mena et al., 2000; Yost & Kelley, 1983]. Hunters are also now hunting species that were previously considered taboo, such as the tapir (*Tapirus terrestris*) [Rival, 1993]. The Waorani maintain a largely traditional lifestyle, but use of forest products has declined, with families increasingly consuming food bought in markets [Franzen & Eaves, 2007]. International interest in the Waorani, Tagaeri, and Taromenane has always been high, but interest in these communities as actors in political and conservation events is likely to increase with the Ecuadorian Government's Yasuní-ITT initiative; the Ecuadorian government is requesting compensation from the international community in return for protecting the Isthpingo-Tiputini-Tambococha (ITT) petroleum block of eastern Yasuní National Park from future oil extraction [Finer et al., 2009].

Previous ethnoprimatological studies in the Amazon have investigated the role of primates in the daily lives of indigenous groups, with particular focus on the relationships with pet, wild and mythical primates, and thus ethnoprimatology *sensu stricto*. Ethnoprimatological research in the Amazon includes case studies of the Tikuna [Parathian & Maldonado, 2010], Guajá [Cormier, [Bibr b6],[Bibr b7]], Bari [Lizarralde, 2002], and Matsigenka [Shepard, 2002] peoples, a review of review of primate consumption and symbolism in lowland South America [Cormier, 2006], and a bibliography of ethnoprimatological resources [Urbani, 2002]. Primates feature in the mythology of many Amazonian peoples, and Cormier [2006] argues that primates in Amazonia perform a special role in helping to define “personhood.” Although it is clear that some Amazonian groups do place great importance on primates in both their material and spiritual lives, there is a lack of information on perceptions of primates in relation to other animals. Investigating the place of primates within the broader ethnobiology of a culture is important, particularly for researchers investigating ethnoprimatology *sensu stricto*. Anthropological studies can reflect the interests and perceptions of the anthropologist as well as the studied culture [Heider, 1988], and most examples of ethnoprimatology *sensu stricto* are based on anthropological methods [e.g., Lizarralde, 2002; Shepard, 2002]. Once individuals in a community are aware of a researcher's interests, they may be more likely to report information on these subjects which, in the case of ethnoprimatology, may create in the researcher an inflated perception of the importance of primates, which may have implications for subsequent conservation efforts.

This study focuses on three small communities in Ecuadorian Amazon, and aims to place primates within the contemporary ethnobiology of the Waorani. Specifically, this study will explore how the Waorani within the study site conceptualize and categorize the group of animals which is known scientifically as the order *Primates*. The relative cultural importance of primates will be measured using freelists to calculate cultural salience. Finally, as other authors have suggested that primates should be culturally important to the Waorani due to the large number of primates consumed, the dietary importance of primates at the study site will be determined.

## METHODS

### Study Communities

Data were collected inside Yasuní National Park, Ecuador, in communities located along the Maxus road, one of two main oil roads entering the Yasuní National Park from the north. The road was built in 1994 and was soon after colonized by people from the Waorani and Quichua ethnic groups. The Waorani communities of Guiyero, Kilómetro 36, and Timpoca participated in this study. The communities are similar to many contemporary Waorani settlements; although they are relatively isolated geographically and rely on traditional subsistence sources of hunting, gathering, and forest gardens, they have frequent contact with outside groups and access to markets, education, and healthcare. An oil company provides a bus service to Pompeya by the river Napo for members of these communities to visit the weekly market, and a bus service at least twice daily during the week to allow children to attend the primary school in Guiyero. The primary school is funded by the company, and the Waorani use the oil company medical centre, located at the nearest large oil extraction facility. There has been no colonization by other ethnicities, except individuals who marry Waorani and come to live in the communities with their spouse. Primates are regularly consumed and frequent pets in these communities.

### Primate Species

Twelve primate species are present in Yasuní National Park, although just ten are present in the study area (Table1). Biological research is conducted throughout Yasuní National Park, but research has primarily been conducted near the two research stations in the north of the park: Tiputini Biodiversity Station (TBS) and Yasuní Research Station (YRS). All primate species in the park have experienced some degree of research, as a result of several long term projects headed by Antony Di Fiore of the University of Texas at Austin on woolly monkeys [Di Fiore, 2004], spider monkeys [Link & Fiore, 2006], noisy night monkeys [Fernández-Duque et al., 2008], red titi monkeys, and saki monkeys [De Luna et al., 2010].

### Interviews

Twenty-seven interviews with 35 Waorani participants from 11 households (a total of 12 households were present in the three communities) were conducted between April and December 2010 (Table2). All willing adult members of the three communities were interviewed, representing 28 of the 39 adults (aged over 16) who were permanently resident during April–December 2010, and three long-term Waorani visitors (resident for longer than 2 months). Four children (aged 12–15) were also interviewed. All individuals in the community were invited to take part in the study. Although no-one directly refused to take part, those who did not participate asked SP to return later when they were free. After two such requests from each individual, they were asked them to contact SP when they were free, as this was understood as an indicator of unwillingness to participate. Eleven individuals did not subsequently contact SP during the study period.

**Table I:** Primate Species Observed at TBS and YRS, Yasuní National Park

Common name	Scientific name
White-bellied spider monkey	*Ateles belzebuth belzebuth*
Poeppigi's woolly monkey	*Lagothrix poeppigii*
Red howler monkey	*Alouatta seniculus seniculus*
White fronted capuchin monkey	*Cebus albifrons aequatorialis*
Common squirrel monkey	*Saimiri sciureus macrodon*
Noisy night monkey	*Aotus vociferans*
Red titi monkey	*Callicebus discolour*
Equatorial saki monkey	*Pithecia aequatorialis*
Pygmy marmoset	*Cebuella pygmaea*
Golden-mantled tamarin	*Saguinus tripartitus*

**Table II:** Number of Participants From Each Community, Divided by Age and Sex

Age and sex of participants	Number of participants from each community			
	Guiyero	Timpoca	Kilómetro 36	Total number of participants
Children aged 12–15	2	1	1	4
Males aged 16–50	6	7	1	14
Females aged 16–50	12	3	2	17
Total	20	11	4	35

Interviews were semi-structured, allowing new questions and topics to be discussed in response to individual responses. Interviews were conducted with single individuals where possible, but on some occasions additional individuals were present and contributed to all or part of the interview. Four individuals who were present but not the intended interviewee gave personal answers about their preferred species and these were included in analyses. For three interviews, two sections of the interview were excluded from analysis (focal animal identification and pile sorting) as multiple individuals were present and it was not possible to assign animal identification to a single individual. Inclusion of these interviews would have created an upward bias in the probability of identification and consumption. As not all individuals who were present answered all questions, sample size varies for each section. All interviews were conducted in Spanish, but on two occasions younger family members were present to act as translators for individuals who did not speak fluent Spanish. All interviews were recorded and later transcribed. Animal names which were not recognized during transcription where identified during informal discussions with informants in December 2010. Spanish words which were unknown were translated by an English speaking Ecuadorian.

### Free Listing

Participants were asked to list the names of all the animals they knew, as a means of placing primates within the broad context of ethnobiological knowledge. Free lists can be used to calculate the cultural salience of named species [Bernard, 2006]. Cultural salience refers to the importance of an item to the culture of a studied community. It is assumed that more important items will be mentioned earlier, and by more individuals during free lists. Salience does not discriminate the “emotional” value attached to any species; species preferred as pets or for consumption may be equally salient as a despised pest. This activity was carried out with all individuals, but the resultant list was only included in the analysis if other individuals present did not contribute to the list, to avoid contamination [Quinlan, 2005]. Eighteen free lists were available for analysis. For animal names in the local language *Wao terero*, the spelling of previous publications has been followed where these were available (authority from Rival [2002] in cases of conflict). For animal names in *Wao terero* for which no previously published record could be found, spelling follows the orthographic rules laid out in Rival [2002, p. xxiii], although some sounds used in animal names were not included in this key. In these cases, spelling followed English spelling rules.

### Identification and Consumption of Specific Species

To assess recognition of key species in the area, and investigate Waorani consumption of these species and their relationship to primates, each participant was shown photographs of 18 common mammal species in the study area (full list in Supplementary Materials). These 18 species included all 10 primate species present in the area and the four most commonly consumed ungulates. The capybara (*Hydrochaeris hydrochaeris*) and tayra (*Eira barbara*) were also included as common mammal species of a similar size which were rarely consumed (according to Franzen [2006]). The kinkajou (*Potus flavus*) and olingo (*Bassaricyon alleni*) were included as previous research suggested these species may be categorized as primates by lowland neotropical cultures [Lizarralde, 2002; Urbani, 2006]. If an individual recognized the animal, they were asked to give its name and whether they had eaten it. During this section, participants gave additional information about the species, and additional questions were asked when appropriate. Decoy primates which were not present in the study area were also included in the set of photographs presented, to validate the assumption that people were indeed aware of the species in their area, rather than guessing. These decoy species were the black and white colobus (*Colobus guereza*) and De Brazza's monkey (*Cercopithecus neglectus*) from east Africa, golden lion tamarin from the Atlantic forest of Brazil (*Leontopithecus rosalia*), and the uakari from Amazonian Brazil (*Cacajao calvus*). The black and white colobus was removed as a decoy species, after it was misidentified as the giant anteater (*Myrmecophaga tridactyla*) in five out of five interviews. This misidentification is likely partly due to the presence of lighter stripes down the torso of both species, and their long tail hair. The remaining three species were stated to be unknown by 20 of 26 participants. One young woman identified the uakari as a spider monkey. However, she also correctly identified the photo of the spider monkey when presented with the image. Six individuals stated they did know one or more of the decoy species, but were not able to name them, because the photo showed a different type of monkey to the species they knew in the area. Decoy species were not included in the analysis.

### Pile Sorting

Free pile sorts are used to investigate how a group of people classify a certain group of objects [Bernard, 2006]. In order to understand whether primates were viewed as a distinct group, and how primates were perceived to relate to other species in the area, participants were asked to sort the 18 species into groups of the animals they thought were similar. Participants were informed that they could group animals in any way they wished. Once informants had finished sorting the photos, they were asked to explain why they had created these groups. Twenty-four pile sorts were conducted.

### Data Analysis

#### Impact of wildlife trade on language use

Information on trade with non-Waorani communities was used to investigate the impact of trade on the language used to refer to the 18 focal species. From January 2005 to May 2007, wildlife passing through Pomeya market was recorded by Suárez et al. [2009], and it is in this market where the Waorani of the study communities sell wildmeat to non-*Wao terero* speakers. The volume of trade for each species at the market was used as a proxy for the likelihood that members of the communities needed to use non-Waorani names for species. A median of two individuals per focal species were observed in the market (range: 0–391 individuals), so species were split into two roughly equal groups; three or fewer individuals observed in the market (11 species), or eight or more observed (7 species).

### Perceived Similarity of Primate and Non-Primate Species

For each of the 18 focal species, the proportion of the 24 pile sorts which placed the species in each of four group types was calculated; in a group with only primate species, in a group with only non-primate species, in a group with primate and non-primate species, or in a group alone. This information was used to examine the perceived similarity of each species to the scientific group Primates.

For each dyad of two focal species (153 dyads in total), the number of pile sorts in which both species were placed in the same pile was calculated. Wilcoxon rank sum tests (identical to a Mann−Whitney *U*-test) were used to compare the number of co-occurrences in a single pile for three types of dyad: primate:primate (p:p), primate:non-primate (p:n), and non-primate:non-primate (n:n). Bonferroni corrections were applied as multiple tests were conducted on the same data set, reducing the significant *P* level to 0.025. Dyads which were placed in the same group in the majority of pile sorts (13 or more) were also identified, and assumed to be perceived as more similar than those which were less often placed together.

### Cultural Salience

Free lists were used to calculate the cultural salience of the animals listed. Calculations of cultural salience have two assumptions; ([Disp-formula m1]) items named by more individuals are more salient (in this case, more central to the concept of “animal”), ([Disp-formula m2]) items named earlier on an individual's list are more salient [Quinlan, 2005]. The following equation (from the calculation method specified by Quinlan [2005]) was used to calculate the salience of each animal mentioned by an individual:

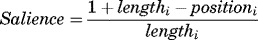
1where *length* is the number of animal names given by individual *i*, and *position* is the location of a specific animal in the list of individual *i*, for example, the first named animal is position 1, the second named animal in position 2, etc. If an animal is not mentioned by an individual, its salience is zero. The cultural salience of each animal is calculated using the following equation:

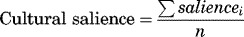
2where *n* is the number of individuals which participated in a study. Cultural salience for each animal named during free listing was calculated using the program ANTHROPAC [Borgatti, 2012]. Multiple names for single animals were grouped for analysis. All participants were to some degree bilingual in Wao terero and Spanish, but Quichua names for animals were also frequently given.

### Dietary Preferences

For each individual, preference scores were assigned to each animal species named as a preferred species for eating, where:

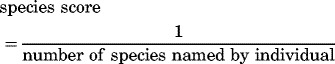
3Therefore, if an individual named a single species when asked which species they preferred, the species was given a score of one. If a species was not listed, it received a score of zero. If an individual named multiple species, each species named was assigned a fraction score. The sum of scores across all individuals was calculated for each species named.

Research plans and protocols were reviewed and approved by the Imperial College Research Ethics Committee (approval reference ICREC_9_2_7) and adhered to the United Kingdom's Animals (Scientific Procedures) Act 1986. Research permit 009-DFO-DPO-M was granted by the Ministerio del Ambiente, Provincial de Orellana, Ecuador to work within Yasuní National Park. Before any interviews were conducted, community meetings were held to introduce the interviewer (S.P.), explain the study purpose, and gain consent from community leaders for the study to continue. Due to low levels of literacy, individuals gave verbal consent to interviews and, after a brief explanation of the study purpose, the interview content and how the information would be used was explained. Participants were informed they were not obliged to participate, and could stop the interview at any point. This research adhered to the American Society of Primatologists principles for the ethical treatment of primates.

## RESULTS

### Species Names and Language Used

**Fig. 2:**
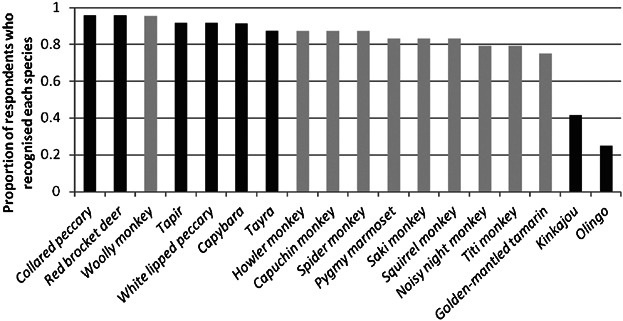
Proportion of 24 participants who recognised and correctly named each of the 18 focal species. Lighter gray bars are primate species. *Potus flavus* and *Bassaricyon alleni* were only considered correctly named when referred to as gamönga and ganata, respectively.

The most recognized species were the white lipped peccary, red brocket deer (*Mazama americana*), and woolly monkey, but all species were recognized by at least two thirds of participants. Nevertheless, some species were frequently confused, in particular, the olingo and kinkajou (Fig. 2). Other species whose names were sometimes confused were the two peccary species, and the red titi monkey and howler monkey. Names for species were given in Spanish, *Wao terero* and *Quichua*, with many participants giving multiple names in different languages for a single species. Nevertheless, all species were most frequently named in *Wao terero*, with the exception of the red brocket deer, which was most frequently named in Spanish, and the white lipped peccary (*Tasyassu pecari*) and collared peccary (*Pecari tajacu*), which were equally likely to be named in *Wao terero* or *Quichua*. The ten species for which three or fewer individuals were traded in Pompeya market were more likely to be referred to in *Wao terero* than those with eight or more records in the market (Wilcoxon rank sum test, *N*_high-trade_ = 7, *N*_low-trade_ = 11, *W* = 68.5, *P* = 0.007).

Participants referred to all ten primate species in the area as *monos* (Spanish, *monkeys*), for example “mono maquisapa” for the spider monkey, suggesting that primates are seen as a group. However, they also referred to the olingo (*B. alleni*) and kinkajou (*P. flavus*) as *monos*. During interviews, participants explained that there were three *monos nocturnos* (Spanish, *nocturnal monkeys*): *gamönga*, *amönka*, and *ganata* (*Wao terero* names). The noisy night monkey was consistently identified as *amönka* by 18 of 19 participants who assigned a name to the photo. Participants were approximately equally divided as to whether the kinkajou or olingo were *ganata* or *gamönga*, though the kinkajou was identified as *gamönga* by nine participants, and *ganata* by four, whereas the olingo was identified as *gamönga* by seven individuals and *ganata* by six, with five participants using the names interchangeably. These inconsistencies in naming the olingo and kinkajou may lie in the difficulties in distinguishing these two species from photos alone—participants stated that the main distinctions were their calls and size, with *gamönga* being bigger, providing further evidence that *gamönga* refers to the kinkajou, which is twice the weight of the olingo. During free listing, the three informants mentioning all three species all referred to them in the order *gamönga*, *amönka*, and *ganata*. One further individual mentioned *amönka*, *ganata*. This highly consistent ordering provides further suggestion that these three species are a culturally cohesive animal group.

### Is the Scientific Family Primates Recognized as an Exclusive Animal Group?

During the pile sorting exercise, two individuals did not make any groups, and stated that all animals were equal and different. Of those who did make groups, two principal explanations for the grouping were given. Firstly animals in a group spend time together and can be encountered together in the forest, or feed on the same foods. Alternately, animals were split into *arriba* (Spanish, *above*, that is tree dwelling animals) and *abajo* (Spanish, *below*, ground dwelling animals, sometimes referred to as *de pata*, Spanish, *of hoof*/*paw*). These individuals also identified a third group, *de pluma* (Spanish, *of feather*), which included all birds. The tayra was identified as a problem animal for categorization by some individuals, as it spent time both in trees and on the ground, and was most often placed in a group alone.

**Fig. 3:**
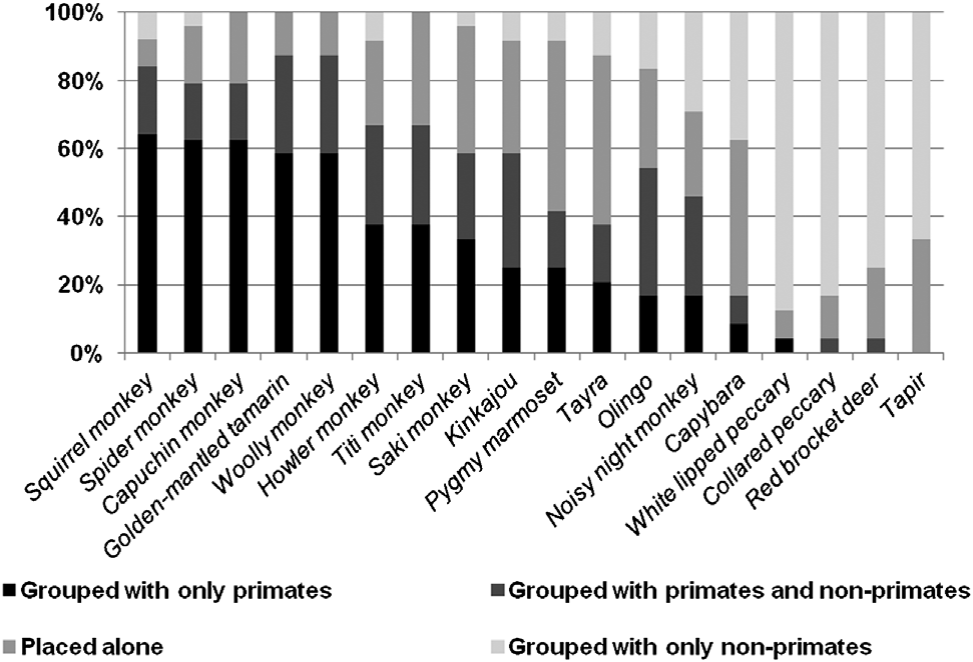
Proportion of pile sorts in which each of the 18 focal species is placed in a group with any primate, placed in a group alone, or place in a group only with non-primate species. Primate species are indicated with an asterisk (*).

During the pile sorting exercise, participants created a median of 5.5 groups (range 2–17, interquartile range), and the ten primates were not grouped together by any individual. The most frequent group given which included any primate was four individuals who grouped the night monkey, olingo, and kinkajou in a unique group. One individual grouped all diurnal primates in a single group. The most common group was the white-lipped peccary and collared peccary, created by nine individuals. Most primates were more frequently grouped with other primates than most non-primate species were (Fig. 3). The pygmy marmoset (*Cebuella pygmaea*) was less frequently grouped with other primates as it was placed in a group on its own in half (12) of the pile sorts. In contrast, the noisy night monkey was less frequently grouped with primates as it was placed in an exclusive group with the kinkajou, olingo, or both in seven pile sorts. These groups with the noisy night monkey also contributed to the high proportion of pile sorts in which the kinkajou and olingo were grouped with primates.

**Figure 4:**
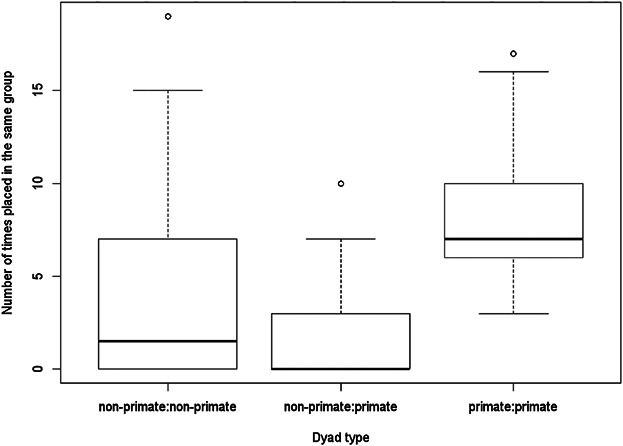
Number of times each dyad type occurred in the same pile during pile sorts.

During the pile sorting exercise, primate pairs co-occurred in a median of 7 piles (interquartile range 6–10). In contrast, the majority of primate:non-primate dyads were never placed in the same pile (median of 0 piles, interquartile range 0–3) which is significantly fewer (Wilcoxon rank sum, *N*_p:p_ = 45, *N*_p:n_ = 80, *W* = 3,484, *P* < 0.001, Fig. 4). The primate:non-primate dyad most commonly placed in the same pile was the noisy night monkey and olingo, placed in the same pile by ten individuals. Non-primate pairs co-occurred in a median of 1.5 piles (interquartile range 0–7), which was not significantly more than the number of piles in which primate:non-primate dyads occurred (Wilcoxon rank sum, *N*_n:n_ = 28, *N*_p:n_ = 80, *W* = 855.5, *P* = 0.047). All primate dyads were placed in the same pile by at least three participants. These results suggest that primates may be viewed as an exclusive animal group. On the other hand, this analysis includes only the subset of species in the area which were included as focal species.

Six species dyads were placed in the same pile by more than half of the participants, suggesting they are generally perceived as similar, but only three of these species dyads were ever placed in an exclusive group by participants:

1. White lipped peccary and collared peccary (19 individuals placed in same group, 9 in exclusive group).
2. Woolly monkey and spider monkey (17 individuals placed in same group, 3 in exclusive group).
3. Squirrel monkey (*Saimiri sciureus*) and golden mantled tamarin (*Saguinus tripartitus*; 16 individuals placed in same group, 0 in exclusive group).
4. Howler monkey and woolly monkey (15 individuals placed in same group, 0 in exclusive group).
5. Red brocket deer and tapir (15 individuals placed in same group, 6 in exclusive group).
6. Howler monkey and spider monkey (13 individuals placed in same group, 0 in exclusive group).

These results suggest that the two peccary species may be seen as a natural and exclusive group—in addition to the pile sorting exercise, qualitative analysis of the interviews showed that seven individuals spontaneously commented on the similarity of the two peccary species, whereas only one participant mentioned similarities between woolly and spider monkeys, and no individual mentioned similarities between the red brocket deer and tapir. The high frequency of grouping of red brocket deer and tapir, in spite of the lack of perceived similarities, may be as both are part of the group of animals “abajo”—along with the two peccary species and the capybara. Six individuals placed these five species in a unique group. Likewise, the three largest primates (howler, woolly, and spider monkeys) could be considered a “fuzzy” group—each dyad of these three species was grouped together by at least half the participants, and although only one individual grouped these three species in an exclusive group, 11 others included the three species in the same group.

### Consumption

**Fig. 5:**
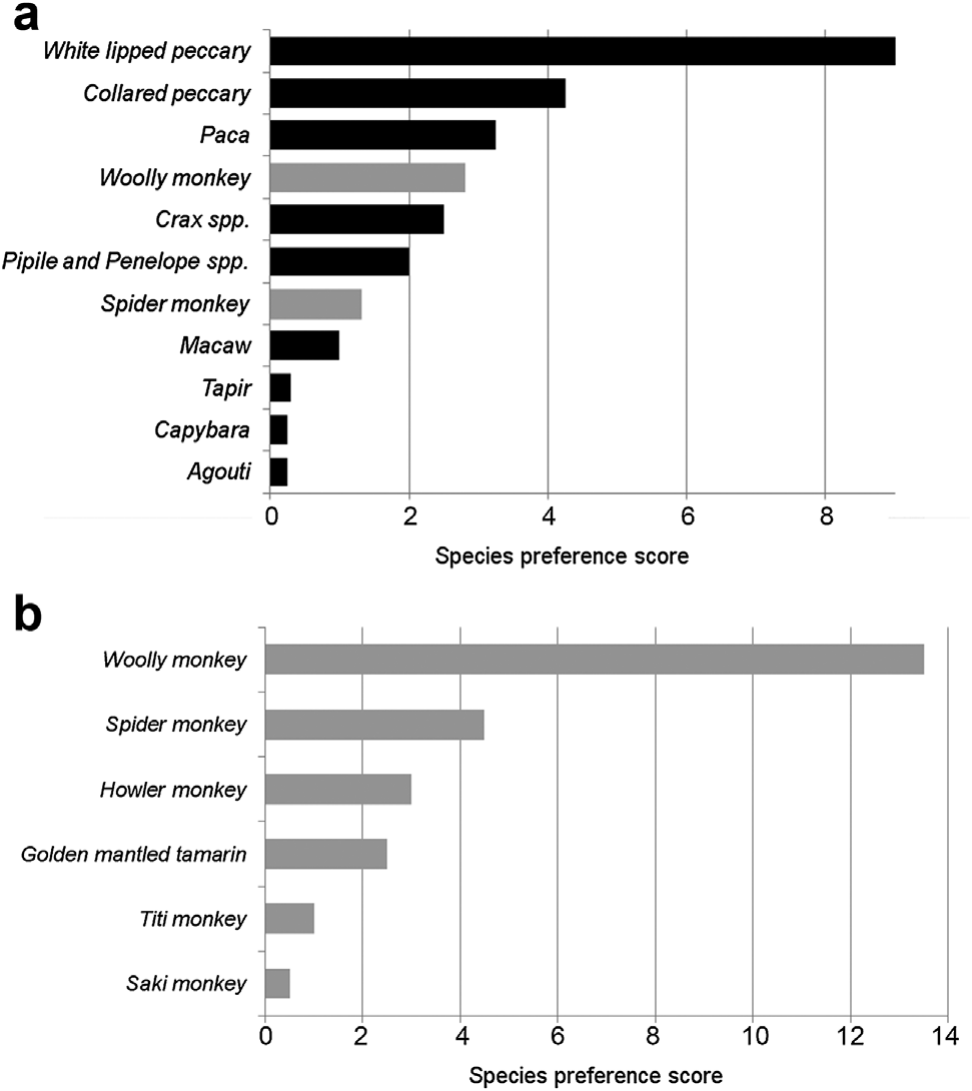
Preferred species for consumption. a: All species, primate species are shown with lighter gray bars. b: Preferred primate species for consumption.

The 27 participants named 12 species as preferred species for consumption, the most popular of which was white lipped peccary (Fig. 5a). The white lipped peccary was also found by Franzen [2006] to be the most important contributor to the diet of the communities when measured by number of individuals and meat weight (45% of all meat weight). When asked about preferences within only monkeys, the overwhelming majority preferred woolly monkeys (preferred species score 13.5 from a sample 24 individuals, Fig. 5b). Individuals named either one or the other peccary species as their favorite, but never named both. In contrast, when asked their favorite primate species, six individuals named both the woolly and spider monkey. It is unclear whether this is because it is difficult to distinguish the two tastes, thus both are equally preferred.

Various taboos were mentioned during conversations with individuals, although some were personal or temporal, rather than prescriptive. Both spider monkeys and saki monkeys were mentioned as species which should not be eaten by pregnant women, with one individual stating that the child will be thin if this happens. Saki monkeys were said to make people ill, as were spider monkeys and the tayra, which gave some individuals headaches and made them feel dizzy. Capuchin monkeys made some individuals tremble. Other individuals also stated they did not like howler monkey, as they had a lot of worms in the meat and tasted bad, but others mentioned howler monkeys as one of their favorite primate meats, suggesting that this was a preference, rather than a taboo.

### Cultural Salience

**Fig. 6:**
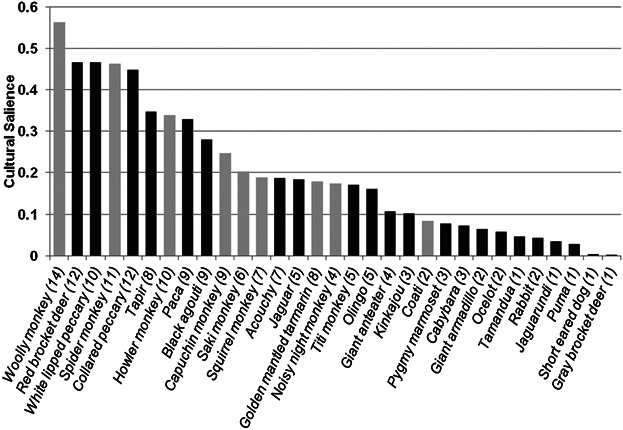
Cultural salience of all mammal species named during free lists by 18 individuals. Lighter gray bars are primate species. Number of participants who named each species is shown in brackets after the species name.

The most salient species were the two peccary species, the red brocket deer and the woolly and spider monkeys. The peccary and monkey species were also preferred species for consumption. Woolly monkeys were the most frequently named species during free listing, and also had the highest salience of any species (Fig. 6). However, the frequency with which individuals named primates, and the mean average position of primates in individuals' lists was almost identical to that of all other mammals, and the mean cultural salience of primates was slightly lower (rank ranges between 1 and 31. Salience ranges between 0 and 1. Primates: frequency = 88.9%, mean rank = 7.88, salience = 0.515. Non-primates: frequency = 88.9%, mean rank = 7.69, salience = 0.575).

### Non-Consumptive Uses of Primates

Woolly monkeys, spider monkeys, owl monkeys, pygmy marmosets, collared peccary, and numerous birds, including macaws (*Ara* species) and Amazon parrots (*Amazona* species), were observed as pets in the communities. During interviews, each focal species was reported to have been kept as a pet by at least one individual, either by themselves or a family member. Golden-mantled tamarins were reported as the preferred species for pets, as they were clean and ate cockroaches, but the most commonly reported (and most commonly observed) pet was the woolly monkey. These pets did not have their own miniature houses, as reported by Mondragon & Smith [1997], but usually lived either inside the house or outside attached to a string. Breast-feeding of these monkeys was neither observed nor reported—most individuals reported feeding their pets cultivated fruits and chicha, a mildly alcoholic drink usually made primarily from yuca. The tails of saki monkeys, squirrel monkeys, and the kinkajou were all reported to be used to decorate traditional crowns. Bird feathers were also used frequently used as decoration, such as the use of *Ara* spp. feathers on hunting spears.

## DISCUSSION

Previous research has emphasized the cultural importance of primates in traditional Waorani society [e.g., Rival, 1993]. Given the extensive changes in Waorani society in the 50 years since first western contact, the results of this study should not be interpreted as representing traditional Waorani perceptions of primates and other animals, but rather as observations on the contemporary ethnobiology of the Waorani. Although the three communities in this study do not represent traditional Waorani society, these results may reflect the ethnobiology of many contemporary Waorani communities which have increased contact with outside groups but still rely on traditional food sources, maintain many features of traditional Waorani culture and are relatively isolated.

All primate species were identified as *monos* by informants and primates were generally more frequently grouped with other primates during the pile sorting exercise. However, the kinkajou and olingo were also referred as *monos*, specifically *monos nocturnos*, and were placed together in a group with the noisy night monkey by a number of informants during the pile sorting exercise. Informants explained this grouping because all three were nocturnal, and lived in the same way; you could find them during the day by banging on hollow trees. This grouping of a primate together with kinkajou and olingo is consistent with naming systems in other parts of the lowland neotropics, where the kinkajou and olingo are referred to as monkeys [e.g., Lizarralde, 2002; Urbani, 2006]. Tayra were also grouped with primate species a number of times. Tayra were seen as a species which transcended the groups of animals “arriba” and “abajo,” as it spent time both on the ground and in the trees, but it was recognized by informants as being different from monkeys as it had “paws.” Other pawed arboreal animals, such as the squirrel, were also never referred to as monkeys. Therefore the grouping of kinkajou and olingo with primates appears in part to be because of their shared space use in the trees, shared nocturnal behavior, use of tree holes as sleeping sites like the night monkey, and because they, like monkeys, have hands rather than paws. This perception of *monos* as a group which includes the kinkajou and olingo may be a consistent pattern of folk taxonomy across the lowland neotropics. Folk taxonomy in Amazonia may be an area for further study, as a clear understanding of how people categorize the animals in their local area can inform conservation. Researchers and conservation practitioners should take particular care that they and those in the studied culture are referring to the same group of animals with the term *monos*, particularly when planning a project with primates. If researchers and local cultures have different ideas about what constitutes the group “primates” or “monkeys,” it may be necessary to determine whether a project should encompass the local or researcher view of this group.

Two species recurrently appeared important: the woolly monkey and white lipped peccary. These two species contributed the greatest number of individuals to Waorani harvest of mammals in the area [Franzen, 2006], were highly recognized by participants, had high cultural salience and were preferred species for consumption. Our results suggest that woolly monkeys are the preferred primate for consumption; other studies have shown that they are also the most consumed primate by the Waorani (Franzen [2006] 42% meat weight of primates; Mena et al. [2000] 22% meat weight of all species). Nevertheless, although woolly monkeys were clearly important to the Waorani, as they were named by most individuals during free listing and had the highest cultural salience of any species, there was no evidence which suggested that primates as a group were more important than other mammal species. On average primates had lower salience than other species; thus in general, cultural importance for the Waorani is better described at the species, rather than order level. Although there was no clear primate group, there was possibly the “fuzzy” group of woolly monkeys, spider monkeys, and howler monkeys, as observed in the Matsigenka by Shepard [2002], who made a convincing argument that this grouping (which in the Matsingenka included the capuchin monkey) was based on the presence of a prehensile tail. This definition of the group would also imply the inclusion of the kinkajou, which has a fully prehensile tail. None of the participants in this study mentioned prehensile tails as a similarity between the three largest primates and the kinkajou and capuchin (though one individual placed these four primates in an exclusive group), suggesting that in the Waorani this “fuzzy” group of the three largest primates may be based on other considerations, such as size or taste preferences. Woolly monkeys and spider monkeys were perceived as the most similar primates—they were often grouped together and some individuals reported both as preferred primate species. One difference between spider and woolly monkeys was in their roles as pets. While woolly monkeys are frequent pets and many individuals spontaneously discussed pet woolly monkeys, discussion of spider monkeys and most other species focused on hunting. It is possible that the woolly monkey has a dual role in the community as pet and prey, similar to the role Cormier [2002, 2003] reports for monkeys in Guajá communities. Alternately, as most of the interviewees knew SP was also collecting data about woolly monkeys [see Papworth et al., 2013], this “importance of woolly monkeys” may be an artefact of the interviewees' perception of SP's interest in this species. Although this is possible, the special role of the woolly monkey is corroborated by previous research on the Waorani by Rival [2002] and Yost & Kelley [1983]. SP was also collecting field data on titi monkeys and no notable differences in participants' attitudes to this species were observed, suggesting that biases caused by SP's other research activities were not the primary reason for the interest in woolly monkeys.

In contrast to monkeys, the two peccary species seemed to form a consistent group. Even though collared peccary were not historically hunted by the Waorani [Yost & Kelley, 1983], the two peccary species were the most preferred species. Being preferred may account for this grouping, but individuals who commented on the similarities between the two peccaries commented on their similar appearance and ecology, rather than a similar flavor. Rival [1996] suggested that the Waorani were not interested in peccary species, and never sought to hunt them. In contrast, she states that the Waorani considered monkeys more interesting; monkeys frequently featured in traditional stories, and men retold stories of hunting trips which encountered monkeys. Although some other primate species made important dietary contributions, had high salience and were also named as preferred species (e.g., the spider monkey), these characteristics cannot be generalized to all primates, and interest in primate in general did not seem any greater than interest in peccaries.

Although most focal species were consistently identified by participants, many had trouble identifying species from photos, particularly the similar olingo and kinkajou, which informants largely distinguished on their size and calls. Although the implications of this confusion were not serious in the current study, multimedia prompts, such as video, or combinations of photos and call playbacks could have improved identification of these species. This observation has particular implications for projects which use photographic methods to identify the presence of specific species. For example, Dechner [2011] used visual prompts to enable local informants to identify forest fragments where the black mantled howler monkey (*Alouatta pallinata*) had been observed. Although 38% of individuals recognized *A. pallinata*, howler monkeys are very cryptic species, and vocal encounters are more frequent than sightings (personal observation). Call playbacks may have increased individual recognition, and identified more areas where the monkeys were present. In a further complication, similar-looking *Alouatta seniculus* also occurred in the study area, and individuals may have confused the two species, or may not view the two as separate species. When attempting to gain information about one particular species using these methods, conservation practitioners should consider including similar sympatric species, to ensure that informants identify the focal species as a distinct animal type, thus ensuring that both the practitioner and informant are discussing the same species.

Ethnoprimatological studies imply, by definition, that primates are particularly worthy subjects of research in the studied culture [Fuentes, 2012], but this assumption needs to be critically examined, particularly in ethnoprimatology *sensu stricto*. By focusing on primates, ethnoprimatologists may overlook other species whose interactions with humans may be important for the conservation of an entire ecosystem. For example, the available evidence suggests peccary species may supersede the importance of primates in the diet of many Amazonian peoples. Although large bodied monkeys and peccaries recurrently feature in the diet of people which rely on wildmeat as a source of protein, many have demonstrated that peccary species are more important than primate species for human consumption [e.g., Cormier, 2003; Franzen, 2006; Parathian & Maldonado, 2010; also in a review of several studies by Sponsel, 1997]. If one of the ultimate aims of ethnoprimatology is to inform conservation, a broader focus on the place of primates in the ethnobiology of a community can only be of benefit. Ethnoprimatologists should take care to place primates within a broader context before embarking on studies focused particularly on primates. Without these precautions, ethnoprimatology *sensu stricto* risks imposing western taxonomic groups on indigenous peoples, rather than allowing them to define themselves which species are important. Even in ethnoprimatological studies which do not involve ethnoprimatology *sensu stricto*, important interactions between humans and other species could be missed by narrowly focusing on primates, which may negatively impact the conservation relevance of the study. In incorporating the wider fauna in a study area, at least during the initial stages of a project, these dangers can be avoided.

## Supporting Information

TABLE SI Focal Species Used for Species Identification and Pile Sorting
