# Supplementary material for: The natural place to begin: The ethnoprimatology of the Waorani
Source: Am J Primatol. 2013 Jul 1;75(11):1117–28. doi: 10.1002/ajp.22173 (PMC4230474; doi:10.1002/ajp.22173)
Supplement: TABLE SI — Focal Species Used for Species Identification and Pile Sorting [file ajp0075-1117-sd1.docx]

**Supplementary materials: Focal species used for species identification and pile sorting**

*Table S1. Focal species used for species identification and pile sorting. The rate of extraction in kg and number of individuals by the communities during 5 months (from Franzen, 2006) and trade at a local market (from Suárez et al. 2009) for each of these species is also shown. Species names follow the IUCN redlist (accessed online 19^th^ June 2012) for both scientific and common names. Primate species are shown in bold.*

| Scientific name | Common name | Name in Wao terero | Other names used by interviewees | Number of individuals extracted | Weight extracted (kg) | Individuals traded at Pompeya market |
| --- | --- | --- | --- | --- | --- | --- |
| *Tasyassu pecari* | White-lipped peccary | Urè | Guangana, pecari de labios blancos | 106 | 2726 | 391 |
| ***Lagothrix poeppigii*** | **Poeppig’s woolly monkey** | **Gata** | **Chorongo, mono, mono lanudo, monkey** | **83** | **471** | **61** |
| ***Ateles belzebuth*** | **White-bellied spider monkey** | **Deye** | **Maquisapa, mono araña, araña** | **58** | **453** | **9** |
| *Pecari tajacu* | Collared peccary | Amo | Sajino, pecari de collar | 28 | 567 | 97 |
| ***Alouatta seniculus*** | **Colombian red howler monkey** | **Iwa** | **Aullador, cotomono, mono colorado** | **28** | **161** | **1** |
| *Mazama americana* | Red brocket deer | Cöhuañe | Venado | 19 | 366 | 49 |
| ***Cebus albifrons*** | **White-fronted capuchin** | **Bogi** | **Machin, mono machin, capuchin** | **18** | **52** | **2** |
| ***Callicebus discolor*** | **Red titi monkey** | **Gänaroca** | **Songosongo, mono titi, cotoncillo** | **10** | **7** | **1** |
| ***Pithecia aequatorialis*** | **Equatorial saki monkey** | **Cuwïncu** | **Parahuaco, mono saki,** | **8** | **21** | **0** |
| ***Saimiri sciureus*** | **Common squirrel monkey** | **Gekirè** | **Bariso, mono ardilla, ardilla** | **4** | **4** | **3** |
| *Tapirus terrestris* | Lowland Tapir | Titë | Danta, tapir | 3 | 352 | 14 |
| ***Aotus vociferans*** | **Noisy night monkey** | **Amönka** | **Mono nocturne** | **3** | **1** | **0** |
| *Eira barbara* | Tayra | Öcata | Cabeza de mate | 2 | 11 | 0 |
| ***Saguinus tripartitus*** | **Golden-mantled tamarin** | **Mimö** | **Chichico, tamarino** | **2** | **1** | **1** |
| *Hydrochaeris hydrochaeris* | Capybara | Tota | Capybara | 1 | 34 | 8 |
| *Potus flavus* | Kinkajou | Gamönga | Ganata, cusumbo | 1 | 2 | 0 |
| ***Cebuella pygmaea*** | **Pygmy marmoset** | **Gatomo** | **Leoncillo, mono de bolsillo** | **0** | **0** | **2** |
